# Transactions of the Medical Association for Southern Central New York, at the Annual Meeting, Held at Cortlandville, June, 5, 1849

**Published:** 1850-03

**Authors:** 


*Transactions of the Medical Association for Southern Central New York, at the annual meeting, held at Cortlandville, June,* 5, 1849. 8 vo. pp. 35.

This Associatiation appears to be sustained with spirit, and the published transactions afford evidence of a degree of zeal and ability on the part of its members, which will ensure its continuance and usefulness. We have transferred to the Eletic department of this number of our Journal, the greater portion of one of the published reports. The Transactions also contain an essay on malarial Remitting fever, by Dr. R. Wilcox. Several other papers of merit were read, the publication of which was not warranted by the limited pecuniary resources of the Association.

The essay by Dr. Wilcox possesses interest, but it appears to us that the Author has not been careful to discriminate cases of Remitting fever, from those of a continued type. In situations where these two forms of fever are presented, they are apt to be confounded, which impairs the value of practical deductions with respect to either form. This is an important subject, respecting which we may offer some remarks at some future time.

We congratulate our friends in Southern Central New York on the success of their organization.

